# Does Practice Make Perfect? The Effects of an Eight-Week Manualized Deliberate Practice Course With Peer Feedback on Patient-Rated Working Alliance in Adults: A Pilot Randomized Controlled Trial

**DOI:** 10.32872/cpe.12353

**Published:** 2024-09-30

**Authors:** Håkan Lagerberg, James F. Boswell, Michael J. Constantino, Gerhard Andersson, Per Carlbring

**Affiliations:** 1 Department of Psychology, Stockholm University, Stockholm, Sweden; 2 Department of Psychology, University at Albany, State University of New York, Albany, NY, USA; 3 Department of Psychological and Brain Sciences, University of Massachusetts, Amherst, MA, USA; 4 Department of Behavioural Sciences and Learning, Linköping University, Linköping, Sweden; 5 Department of Biomedical and Clinical Sciences, Linköping University, Linköping, Sweden; 6 Department of Clinical Neuroscience, Karolinska Institute, Stockholm, Sweden; Philipps-University of Marburg, Marburg, Germany

**Keywords:** deliberate practice, working alliance, cognitive behavioural therapy, professional development, psychotherapy outcomes, therapist effects

## Abstract

**Background:**

Deliberate Practice (DP), which underscores the importance of expert mentorship, personalized learning objectives, feedback, and repetition, has been suggested as a method to enhance the effectiveness of therapists.

**Method:**

The study tested the efficacy of an eight-week, structured, group-based online course, enriched with peer feedback, for 37 Cognitive Behavioral Therapists. The goal was to assess whether this intervention could boost the quality of therapist-patient alliances, as compared to a control group. To measure this, therapists had their patients anonymously fill out the Session Alliance Inventory both before and after the course. The trial encompassed 120 patient alliance ratings at baseline and 64 at the post-course measurement. The DP course was comprised of a 75-minute remote video workshop each week for eight weeks, supplemented by related study materials. Each workshop focused on a specific skill, such as responding to client resistance, and included 55 minutes of concentrated role-play activities, providing ample opportunities for repetition and feedback.

**Results:**

Using a linear mixed model we did not find an effect on patient alliance ratings. However, we observed a trend (*p* = .054) indicating that the DP group decreased their alliance ratings (Cohen’s *d* = -0.40), while the control group demonstrated an increase in their scores (*d* = 0.49).

**Conclusion:**

This pilot study did not find support for DP leading to better patient-rated alliance compared to a waitlist control. However, the study had several methodological limitations. Further and more rigorous investigation of the effects of DP on patient outcomes is recommended.

## Highlights

- An eight-week deliberate practice course did not significantly improve patient-rated therapeutic alliance.
- An unexpected trend showed that deliberate practice training decreased alliance ratings, while the control group showed improvement.
- Peer feedback without expert mentorship may limit the effectiveness of deliberate practice courses.
- Further research on deliberate practice's effects is needed, focusing on individualized learning objectives and expert mentorship.

## Background

Contrary to what might be expected, emerging evidence indicates that therapists do not necessarily improve their psychotherapeutic outcomes with increased experience, defined as accumulation of time in routine clinical practice. For example, in a large-scale longitudinal therapist professional development study using data from more than 150 therapists and 6,500 patients, Goldberg et al. (2016) found that therapists overall became slightly less effective over time.

Consequently, establishing new tools for developing expertise over time appears to be in the interest of the field of psychotherapy. To this end, Deliberate Practice (DP) is emerging as a tool for psychotherapists to continually improve their therapy outcomes (Boswell et al., 2020; Miller et al., 2020; Rousmaniere et al., 2017; Wampold et al., 2019). The effectiveness of DP in the acquisition and refinement of skill has been demonstrated in athletics and music (Ericsson & Pool, 2016), and is now being applied to therapist development. Miller et al. (2017) summarized four central elements of DP for psychotherapists: 1) a focused and systematic effort to improve performance pursued over an extended period; 2) involvement and guidance from a coach/teacher/mentor; 3) immediate and ongoing feedback; and 4) successive refinement and repetition via solo practice outside of performance.

One proposed method of applying DP with therapists is to practice therapy skills using vignette-based role-play (Vaz & Rousmaniere, 2022). To this end, a series of practice manuals for different psychotherapy orientations have been published, including emotion-focused therapy (Goldman et al., 2021), cognitive behavioral therapy (CBT; Boswell & Constantino, 2021), motivational interviewing (Manuel et al., 2022), systemic family therapy (Blow et al., 2023), and child and adolescent psychotherapy (Bate et al., 2022). The manuals include method-specific skills (e.g., working with cognitions in the case of CBT) and several method-non-specific skills (e.g., responding to client resistance). Each skill is described, and skill criteria are provided, with the manuals’ emphasis placed on client vignettes for therapists to role-play and actively work with feedback from expert supervisors or other trained peers. To our knowledge, the effects of these manuals on therapy outcomes have not yet been empirically investigated.

Although the research on the role of DP in psychotherapy is still in its infancy, some progress has been made. Chow et al. (2015) found that the time spent engaged in DP activities predicted therapist-level treatment outcomes across 1,632 patients and 17 therapists. However, the variance explained by DP was extremely small (0.3%) and the retrospective DP self-rating instrument used has been criticized because it relies on therapists’ retrospective recall and the ability of therapists to accurately differentiate DP from other forms of practice (Clements-Hickman & Reese, 2020). The study did not report participants theoretical orientation. Also, Janse et al. (2023) were not able to replicate the findings in the previously mentioned study. In an experimental study, Westra et al. (2021) found that participants randomized to a DP workshop were significantly more likely to respond effectively to client resistance when compared to participants in a traditional workshop, both at post-workshop and at a three-month follow up. However, the study involved simulated patients and did not include any real patient data. Participants reported several primary theoretical orientations.

Hill et al. (2020) reported a single-case study on seven psychodynamically oriented doctoral students who participated in an eight-hour workshop and four individual DP training sessions with in-between session homework. They found that the DP training improved the students’ self-ratings of their knowledge of the practiced skills, emotional self-regulation, countertransference, and working alliance. However, there was no significant effect on the client-rated working alliance. Perlman et al. (2020) completed a randomized controlled trial where therapists who participated in a DP workshop scored higher on facilitative interpersonal skills than therapists who underwent a traditional workshop (Anderson et al., 2009). The therapists reported several primary theoretical orientations. Interpersonal skills were measured using an instrument that has been found to predict therapy outcome (Anderson et al., 2016). In line with previous research, the study involved simulated patients and did not include real patient data which could be viewed as a limitation. Goldberg et al. (2016) completed a study at a Canadian health agency that applied DP and routine outcome monitoring (Lambert & Harmon, 2018) over seven years, including over 5,000 patients and 135 therapists. The intervention achieved an increased effectiveness, as per the routine outcome tool, of *d* = 0.035 per year, reflecting a compounded increase of *d* = 0.25 over seven years. However, the study had no control condition and combined routine outcome monitoring with DP, preventing us from isolating the effects of DP.

In conclusion, although DP has been demonstrated to have a positive effect on some factors related to patient outcomes (including simulated patients), there is, to date, paucity of evidence that DP directly influences patient-rated outcomes. This was also the conclusion of the systematic review of the research on DP, conducted by Nurse et al. (2024). In their review, they emphasize the lack of studies investigating the impact of DP on client outcomes as a main limitation in the literature and encourage studies exploring the impact of DP on client outcomes in actual practice settings. For example, the study by Hill et al. (2020) found that after DP, therapists increased their self-rated efficacy and therapist rated alliance but found no increase in patient-rated alliance.

The current study attempted to bridge this gap in the literature, using working alliance as a patient-rated outcome. There is robust evidence for a moderate correlation between alliance and therapy outcome (*r* = .28, Flückiger et al., 2018). For many years, whether alliance leads to symptom reduction or vice versa has been up to debate. However, a recent meta-analysis found empirical evidence that alliance and symptom reduction have a reciprocal relationship, predicting each other, at least in the early stages of treatment (Flückiger et al., 2020). Also, working alliance has been suggested as one of the mechanisms through which DP could improve therapy outcome (Miller et al., 2020).

In the current study design, using working alliance ratings enabled the researchers to collect within-therapist data at the pre- and post-measure, while collecting cross-sectional data on the patient-level. In order to use symptom outcomes, longitudinal within-patient data would have been necessary, which was not in the scope of the current study. The current design made it possible to recruit therapists from a multitude of psychotherapy settings, increasing the ecological validity. The authors recognize that in this study, working alliance acts as a proxy for patient therapy outcomes, and see this as a major limitation. However, we believe that this study is a step forward for the research literature on DP in moving towards patient-level data. Given the recently published DP manuals which allow for the use of standardized and approved working materials and methods, the present pilot study examined whether patients of therapists who participated in an eight-week DP course improved their patient alliance ratings compared to patients of therapists in a waitlist control group. Based on the results of Perlman et al. (2020) and Westra et al. (2021), along with the literature on skill development and DP for therapists, we expected that the DP group would improve their patient-rated alliance measures compared to the waitlist control group.

## Method

### Study Design

The pilot study used a randomized parallel-arm controlled trial design, allocating the therapists to either receive the DP course or a waitlist control group. The study was conducted in Sweden, and therapists and patients were recruited nationally during January 2022. The study was approved by the Swedish Ethical Review Authority (ID: 2021-05913-01).

### Outcome Measurements and Data Collection

Therapeutic alliance was the primary outcome measure assessed using the Session Alliance Inventory (SAI; Falkenström et al., 2015). The SAI is a brief patient-rated instrument consisting of six items that measure the client’s experience of the alliance during the past session on a scale from 0 (“Not at all”) to 5 (“Completely”). The SAI is a shortened version of the Working Alliance Inventory (WAI; Horvath & Greenberg, 1989), which is a common instrument for measuring therapeutic alliance. The SAI correlates highly with the WAI (*r* = .91; Falkenström et al., 2015) but takes much less time to complete, potentially resulting in fewer missing data. In the current study, we observed an internal consistency of α = .87 at the baseline measurement and α = .85 for the post-measure.

### Procedure and Participants

Swedish CBT-therapists interested in receiving DP training were recruited through professional online forums and the listserv for members of the Swedish Association for CBT and the Swedish Association for Behaviour Therapy. The inclusion criteria were having received a Swedish undergraduate diploma in CBT (involving approximately a minimum of four years of full-time studies and 2 years of clinical practice), currently providing individual CBT for adult patients, being able to commit to 75 minutes of DP weekly for the duration of the program, and being able to recruit patients for the study.

**Figure 1:**
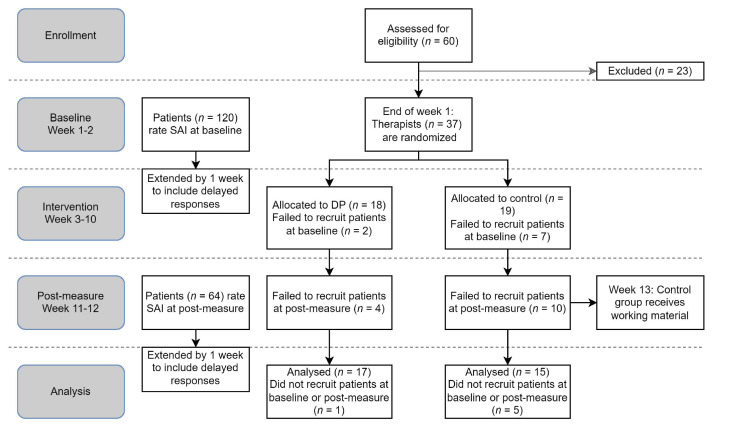
Study Procedure

A total of 60 therapists initially applied (see Figure 1), of which 37 were included and subsequently randomized. Of the 23 excluded participants, 15 did not complete the initial submission form or did not confirm their participation, and eight did not meet the inclusion criteria, including not having the required credentials, not having enough time or enough clients. See Table 1 for the therapists’ sociodemographic information and professional backgrounds. Since the therapists worked in different organizations, therapy was delivered in a number of settings, both privately and community financed. The therapists were asked to report previous experience with DP. In the DP-group, one therapist reported previous experience with Feedback-Informed Treatment and having attended a conference on DP. One other therapist had attended a single lecture on DP. In the waitlist, one reported having attended a workshop in DP, one reported no training but significant interest in DP and one reported having a supervisor who was influenced by DP.

**Table 1:** Sociodemographic Background for the Therapists at Baseline

Sample characteristics	Deliberate practice (*n* = 18)	Waiting list (*n* = 19)	Total (*n* = 37)
Gender, *n* (%)			
Female	13 (68.4)	15 (78.9)	28 (75.7)
Male	5 (26.3)	4 (21.1)	9 (24.3)
Age			
*M* (*SD*)	40 (12.5)	44.7 (9.0)	42.4 (10.9)
Min-Max	26 – 64	26 – 58	26 – 64
Years clinical experience			
*M* (*SD*)	10.3 (9.2)	11.8 (6.2)	11.1 (7.8)
Min-Max	1 – 26	1 – 22	1 – 26
Treatment sessions/week at baseline			
*M* (*SD*)	17.2 (12.3)	18.2 (7.9)	17.7 (10.1)
Min-Max	1 – 45	5 – 30	1 – 45
Therapy setting, *n* (%)^a^	*n* = 19	*n* = 20	*n* = 39
Primary Care	4 (21.1)	5 (25.0)	9 (23.1)
Psychiatry	6 (31.6)	4 (20.0)	10 (25.6)
Private Practice	5 (26.3)	6 (30.0)	11 (28.2)
Privately owned clinic	4 (21.1)	4 (20.0)	8 (20.5)
Other	0 (0)	1 (5.0)	1 (2.6)

^a^Participants working in multiple organizations have been counted multiple times. The percentages are calculated based on the number of work contexts, not the number of individuals.

Patient recruitment took place during a two-week period before and after the DP course. All therapists were instructed to recruit all of their patients who fulfilled the inclusion criteria: at least 18 years of age and receiving individual CBT. The therapists were told to briefly describe and supply the patients with written information about the study and to obtain informed consent. The therapists were told to clarify that participation was completely anonymous and voluntary, that the decision to participate or not would not have any consequences for the patient, and that the therapist would not be able to access their patients’ ratings or even see if the patient joined the study. Video and telephone sessions were included but text-based or internet-only delivered treatments were excluded.

Patients who agreed to participate in the study used a web link or QR code to access the research platform Iterapi (Vlaescu et al., 2016), where they read about the study and provide informed consent for anonymous participation. They then completed the SAI and submitted their therapist’s name. For privacy and ethical reasons, no information about the patients was recorded.

Therapists were instructed to collect data from every patient they had a therapy session with, irrespective of diagnosis, how long the patient had been in therapy, or if they had or had not previously participated in the study. This means that some patients may have participated multiple times during the same measurement period, and that some participated only during baseline, some only during post-measure and some at both measurement points. Because the patients participated anonymously, we were not able to follow the patients over time—only the therapists.

The patient recruitment procedure resulted in the researchers having no information about the diagnosis status, severity, previous therapy experience or demographic data from the patients. This was partially for privacy and ethical reasons, but also a careful decision to prioritize making participation extremely brief, allowing for as many participations as possible. Exploration of the effects of these variables was not in the scope of the current study.

After the first week of recruiting patients, the therapists were randomized to a DP course (*n* = 18) or waiting list (*n* = 19). For the allocation of participants, a fellow MSc student who was not involved in the study used Random.org, a web-based automated randomization service. Once the recruitment period was terminated, the DP group entered the DP course.

When all the data had been collected, we observed that a significant number of SAI ratings had been reported during the week after both measurement periods. We interpreted this as patients remembering to participate the week after they were formally invited, and thus extended both data collection periods by one week to include the delayed patient ratings. Because of this delay, it is possible that some of these patient-ratings were completed after the therapist had participated in up to two DP-sessions.

### Deliberate Practice Course

The DP course was inspired by *Deliberate Practice in Cognitive Behavior Therapy* (Boswell & Constantino, 2021) and was facilitated by the first author of this paper, a master’s student in Stockholm University’s clinical psychologist program. Throughout the DP course, the facilitator consulted a certified DP coach who was a licensed psychologist and psychotherapist. The DP coach cofacilitated one of the DP sessions. The participants had online access to translated working materials for each session, consisting of a brief introduction to the skill being practiced, skill criteria, and client vignettes. The work material was produced by Boswell and Constantino (2021). In line with Westra et al. (2021) and Perlman et al. (2020), we designed the DP course to be group-based for two reasons. First, we wanted to recruit enough therapists to perform statistical analyses. Second, a group-based format improves the affordability of and access to expert mentors. Accordingly, the DP course was offered online in its entirety using digital meeting software and an online platform. Each of the eight weekly sessions was 75 minutes long and consisted of a short introduction to the skill to be practiced, followed by 55 minutes of role-plays and concluded with a few minutes to reflect. During the role-plays, the participants were divided into groups of two or three in separate breakout rooms. They took turns playing the roles of client, therapist, and observer. The client role-played the vignettes, and the therapists attempted to give an authentic response in line with the skill criteria being practiced. See Table 2 for the vignettes and example responses from the working manual. The participants did not have access to example responses during the practice sessions, as we assumed that this would have hampered the authenticity of their responses. After this, the observer and the client gave feedback to the therapist based on the skill criteria and their own observations. After receiving the feedback, the process was repeated, maximizing repetition and feedback exposure for the therapist.

**Table 2:** Examples of Client Vignettes and Example Therapist Responses (Boswell & Constantino, 2021)

Skill	Client Vignette	Example Therapist Response
**Working with behaviours**	[*frustrated*] I don’t know why I keep blowing up at people. I just do.	Let’s try to understand this together. Understanding your response in context can help us achieve some clarity. Let’s start with a recent example and try to identify what was happening just before the “blow up”.
**Responding to client resistance**	[*pessimistic*] I know I agreed to this approach, and I understand what we’re trying to do here, but I’m starting to doubt it’s a good fit.	I’m so glad you told me this, as your outlook on therapy is central to it working. Let’s shift gears for a moment, put aside our agenda, and just discuss what has or has not felt like a fit for you. How does that sound?

Each role-play was very brief and was terminated after the therapist had given their response to the vignette. This approach of using very short sessions is a hallmark of DP role-play (Vaz & Rousmaniere, 2022), as it better allows for detailed feedback and repetition compared to longer, improvised dialogues. Between role-plays, the therapist completed a reaction rating of how challenging they had perceived the vignette. This rating guided the choice to make the next role-play easier or more difficult. For this reason, all vignettes were assigned difficulty levels. Also, the participants were instructed in methods to decrease or increase the difficulty of the vignettes, such as by modulating their level of affect. The groups worked with each vignette until they felt they had exhausted it, between one and six repetitions. The participants switched roles to allow all participants to practice during every session. The facilitator alternated between the groups, giving them feedback and support and answering questions.

The manual published by Boswell and Constantino (2021) instructs facilitators that the role-plays should be completed by two trainees and one supervisor with training in DP, where the supervisor is the one giving feedback. This study used peer feedback instead, allowing for a much larger number of participants, at the potential expense of the feedback quality and the facilitator’s ability to model responses. To ensure the study's focus and feasibility within an eight-week timeframe, a deliberate selection of skills was necessary from the comprehensive set outlined in the manual by Boswell and Constantino (2021), which details 10 core skills for effective CBT practice. Given the pilot nature of this study and the constraints associated with an intensive, focused training program, we prioritized skills that we hypothesized would have the most immediate impact on enhancing the therapist-patient working alliance - our study's primary outcome measure. Consequently, we included seven skills, with particular emphasis on “responding to therapeutic alliance ruptures” by allocating two sessions to this area. This emphasis aligns with literature suggesting the pivotal role of managing alliance ruptures in therapy outcomes.

The skills “explaining the treatment rationale for CBT”, “negotiating a session agenda”, and “adherence flexibility” were excluded. While these skills are fundamental to CBT and contribute to comprehensive therapist training, our decision to exclude them was twofold. First, it was based on the practical need to adapt the extensive content of the manual to a manageable scope that could be effectively covered within the limited duration of our intervention. Second, considering the advanced training level of participating therapists and the study's specific focus on the working alliance, these skills were assessed to be less immediately relevant to the pilot study's objectives. This strategic exclusion allowed for a concentrated exploration of the selected skills, facilitating depth of learning and practice within the study's timeframe.

This selection process reflects a strategic decision-making framework aimed at optimizing training effectiveness by focusing on skills with direct implications for our research objectives, within the practical constraints of an eight-week training program. Such decisions are essential for the design of focused, feasible, and impactful training interventions in research settings.

### Waitlist Control Group

Therapists that were randomized to the waitlist control group were informed that they had been allocated to the waitlist control group and that they would receive the study material at the end of the data-collection period. No blinding or control intervention was used. After the second data-collection, they received text and video-based material from the DP course curriculum.

### Analyses

Linear mixed models (LMMs) were chosen for the analysis. LMMs allow for missing data points without listwise exclusion, allowing the principles of *intention to treat* to be followed. However, LMMs assume that the data are missing at random, which is a potential weakness. The modeling was performed in IBM SPSS Statistics for Macintosh, ver. 27.0, using the MIXED command. A restricted maximum likelihood was used as the sample was small (Luke, 2017). Fixed variables in the final fitted model were the main effects of time and group, as well as the interaction effect of time (pre- and post-measurement) and group (randomization to DP or waiting list). The covariance structure was set to unstructured. Assumptions were tested and data were found to be suitable for the planned analyses.

## Results

The therapists had difficulty recruiting patients for the study. Of the 37 therapists, 29 managed to collect data during baseline, whereas only 23 collected data at post-measure. Failure to collect data was greater in the control group; at baseline, seven (36.8%) participants failed to recruit any patients, growing to 10 (52.6%) at post-measure. The DP group had fewer therapists who failed to recruit any patients: two (11.1%) at baseline and four (22.2%) at post-measure.

During the baseline measurement, a total of 128 session-level patient-reported alliance ratings were obtained for the 29 therapists with baseline data. Of these, we were unable to match eight ratings to a therapist, resulting in a mean of 4.14 ratings per therapist. At post-measure, only 71 session-level patient-reported alliance ratings were collected for the 23 therapists with post-measure data, of which seven ratings could not be matched to a therapist, resulting in a mean of 2.78 ratings per therapist. The variance between therapists was significant; during baseline, therapists collected between 1–12 ratings, and the corresponding number for the post-measure was 1–14.

**Figure 2:**
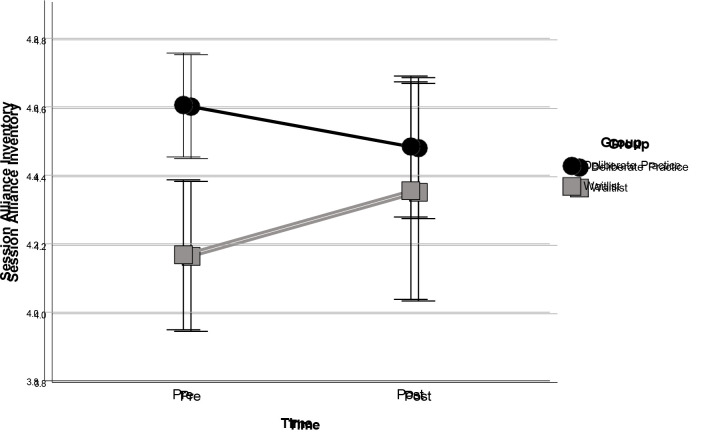
Change in Composite Scores on the Session Alliance Inventory at Baseline and Post-Measurement *Note.* Error bars represent 95% CI.

Results from the LMM showed that the interaction effect of time and group was not statistically significant (*F*_1,19.336_ = 4.208, *p* = .054). However, we also conducted a visual inspection of the data and explored within-group effect sizes to better understand potential training effects. Visual inspection of Figure 2 and pairwise comparisons revealed that allocation to DP had a decreasing effect on patient-rated alliances, while allocation to the waiting list had an increasing effect on SAI scores. The within-group effect sizes were moderate but in opposite directions for both conditions (*d =* -0.40 for the DP group and *d* = 0.49 for the control group).

## Discussion

This pilot study tested whether an eight-week role-play-based online course in DP for CBT therapists would increase therapist-level therapeutic alliance quality in a naturalistic psychotherapy setting. The LMM did not find a significant effect of randomization to the DP course (*p* = .054). Contrary to expectations, we observed a trend (*p* = .054) indicating that the average patient-rated alliance in the DP group decreased (*d* = -0.40) whereas the waiting list’s average patient-rated alliance increased (*d* = 0.49). The study had several limitations, such as missing data, risk that the therapists were biased in their recruitment of patients, and considerable attrition. The results should be interpreted with caution. To our knowledge, this is the first empirical study on DP for therapists to report potential negative effects. However, DP is a broad term, and this study only tested one specific approach to performing DP. The conclusions may not apply to the full manual or to other methods of executing DP. Therapy works through a multitude of mechanisms that are still not well understood (Cuijpers & Cristea, 2016). This fact, combined with the methodological issues previously mentioned, cause us to believe that speculation on specific mechanisms as to why the DP-groups alliance ratings did not improve, although thought-provoking, is of lesser importance, instead, the discussion will focus on confounders for future studies. In the DP course, expert mentorship was not applied which is a central tenet of DP (Ericsson et al., 1993; Miller et al., 2017). However, the original definition referred to “a teacher in a domain with a well-developed knowledge about effective methods for improving aspects of performance” (Ericsson & Harwell, 2019, p. 5). Currently, uncertainty exists as to whether psychotherapy can be defined as such a domain, as evidenced by Goldberg et al. (2016). Nevertheless, the current study relied on peer feedback rather than expert mentorship. The mentor’s primary tasks in this format were to give brief feedback and to help the participants stay in the optimal learning zone by adjusting the difficulty—not too easy, not too difficult. The participants did not have any training in these specific DP coaching skills. The results of the study may indicate that an expert mentor is required for this form of DP to be effective, thus impeding the prospect of scaling up DP courses by increasing the ratio of participants to mentors.

A second tenet of DP is individualized learning goals (Ericsson et al., 1993). This study used standardized vignettes and skills, adjusting the vignettes’ difficulty levels based on the previously described reaction ratings completed by the participants. Participants may have spent time practicing skills at which they were already adept, resulting in no change, or practicing skills that were too difficult, which could lead to shame and withdrawal from the exercise and, ultimately, reduced self-efficacy. Assessments of skills and weaknesses made by an expert or through other means may be crucial for the development of expertise through DP. The chosen skills may have been too demanding, leading to some of the aforementioned adverse effects.

Surprisingly, the control group increased their alliance ratings. Asymmetrical dropout could reflect underlying differences between therapists, such as interest in DP or caseload and thus caused a selection bias. Furthermore, therapists in the control group, as in the DP group, may have been biased in some other way when recruiting patients.

The finding that a manualized DP course with peer feedback led to no change in patient-rated alliances is noteworthy. Therapy is a complex interpersonal process where the same actions can lead to wildly different results, while DP is a reductionistic activity that was developed for mechanistic tasks, such as playing the instruments or typing (Clements-Hickman & Reese, 2020). There is growing evidence that the variance in expertise or performance explained by DP may vary greatly by domain (Hambrick et al., 2016). Although the study by Chow et al. (2015) is frequently cited as an argument for the importance of DP, DP only explained 0.3% of the variance in performance, which paradoxically bolsters the argument that DP may play a smaller role in psychotherapist improvement than in other domains. Previous studies have identified that specific psychotherapy-related skills can be trained through DP (Perlman et al., 2020; Westra et al., 2021), but it remains uncertain whether these relatively simple skills translate to actual symptom improvement in patients.

DP is an attractive method because it initially seems very logical: “Practice makes perfect.” However, the criteria for the original definition of DP are difficult to attain (Ericsson & Harwell, 2019), and practice that does not meet these criteria may not yield the same effects as DP. At the same time, excessively strict boundaries around DP may impede innovation and advancements specific to the setting of psychotherapy (Nurse et al., 2024). We argue that the DP course applied in the present study is an example of attempting to scale up DP by standardizing and reducing its complexity. Future attempts to confirm or disconfirm the effectiveness of DP in the psychotherapy domain should adhere to the original definition and/or to particular modes of application (e.g., more individualized focus, or reliance on an expert supervisor rather than a peer). As Goodyear and Rousmaniere (2017, p. 84) wrote, “Practice makes permanent, though not necessarily perfect.”

The current study had several limitations. In the following section, limitations will be reviewed.

The therapists recruited their own patients. Estimates suggest that SAI-ratings were collected for about 10% of sessions during baseline and for about 6% during post-measure. This introduces a risk of bias. However, the data were collected anonymously via an encrypted website, and since no personal information was collected regarding the client, identification was impossible, reducing the risk that patients would inflate their alliance ratings due to social desirability. We do not have any data to explain the small percentage of recruited patients but speculate that it was caused by forgetfulness or lack of motivation on the therapists’ end, which would explain the drop in participation at the post-measure, as well as the control group recruiting fewer patients. There was asymmetrical attrition in the studied population due to difficulties recruiting patients. Especially in the control group, five (26.3%) participants failed to recruit any patients during baseline or post-measure. Furthermore, some therapists only recruited a single client, potentially causing issues with the LMM.

The study used patient-rated alliance as the primary variable. Preferably, a patient-rated symptom scale would have been used, as it is possible that DP works through mechanisms other than alliance. This choice is further discussed in the introduction of the current article. The authors encourage future research to use symptom rating scales. Of course, this necessitates using longitudinal within-patient data, which was unfortunately not possible with the current data set, where patients for privacy and ethical reasons, participated completely anonymously. Within-patient data would enable researchers to use more powerful statistical analyses, such as multilevel modelling. Also, it was not possible to include session number in the analysis, a factor that predicts alliance (Meier & Feeley, 2022). We suggest that future studies include within-patient data.

A recent meta-analytical finding is that alliance measures are prone to moderate to large ceiling effects. There is a current debate as to whether these ceiling effects are caused by methodological issues or theoretical factors (Meier & Feeley, 2022). It is unclear as to what effect this may have had on the current study.

The manual used was not explicitly focused on therapy alliance, but rather on overall therapist effectiveness. It is possible that a DP manual that more explicitly focused on alliance would have had a greater impact on this variable. However, several chapters focused specifically on alliance-related therapist behaviors.

The study used a waitlist control group. In clinical psychological research this has been shown to inflate effect sizes of treatment groups (Patterson et al., 2016). However, in this study the patients did not have to wait, only the therapists, so it is unclear whether the natural worsening effects that have been observed would apply. It could however explain the greater attrition in the wait-list control. A superior control condition would have been a placebo course as was employed in the excellent study by Westra et al. (2021).

Finally, longitudinal data tends to be underpowered when only two time points are used. Follow-up data would have increased the power of the study.

### Conclusions

This pilot study did not find a significant effect of a CBT-focused online group and manual-based DP course with peer-feedback on patient-rated alliance. However, a non-significant negative effect of DP on patients’ alliance ratings was observed. Although the study had multiple limitations such as considerable asymmetrical attrition, missing data and risk of bias in the data collection, we found the results surprising enough to publish. The current study is also a step forward towards studying direct client outcomes, which has been missing in the literature on DP (Nurse et al., 2024), and may serve as a foundation for future research. As DP is becoming an increasingly popular tool for therapist development, testing the effectiveness of the method is crucial. The authors conclude that future research should adhere more strictly to the original definition of DP and include patient outcomes.

## Data Availability

Although the principles of Open Science guide our work, the sensitive nature of the data collected in this study precludes its open online availability. However, we fully support scholarly inquiry and collaboration. Researchers interested in accessing the data may contact us directly. Measures to protect participant privacy will be maintained during any data sharing.

## Preregistration

This study was not pre-registered. The hypotheses, methodology, and analyses were developed before data collection; however, they were not officially recorded on a public preregistration platform prior to the commencement of the study. Despite this, we have made efforts to conduct this research with transparency, scientific rigor, and integrity.

## Reporting Guidelines

The methodology, results, and interpretation of this study have been shaped and rigorously guided by the Consolidated Standards of Reporting Trials (CONSORT) guidelines, enhancing the transparency, reliability, and replicability of our research findings.
